# Training of adult psychiatrists and child and adolescent psychiatrists in europe: a systematic review of training characteristics and transition from child/adolescent to adult mental health services

**DOI:** 10.1186/s12909-019-1576-0

**Published:** 2019-06-13

**Authors:** For the Milestone Consortium, Swaran Singh, Helena Tuomainen, Jason Madan, Jane Warwick, Cathy Street, Dieter Wolke, Moli Paul, Priya Tah, Rebecca Appleton, Alastair Canaway, James Griffin, Giovanni de Girolamo, Giulia Signorini, Paramala Santosh, Ilyas Sagar-Ouriaghli, Natalie Heaney, Diane Purper-Ouakil, Frédérick Russet, Virginie Maurice, Véronique Humbertclaude, Athanasios Maras, Larissa van Bodegom, Mathilde Overbeek, Esther Kooymans, Ulrike Schulze, Melanie Saam, Ulrike Breuninger, Sabine Tremmery, Gaëlle Hendrickx, Fiona McNicholas, Aleksandra Gronostaj, Tomislav Franić, Nikolina Davidović, Frank Verhulst, Gwen Dieleman, Suzanne Gerritsen, Kate Lievesley, Amanda Tuffrey, Anna Wilson, Charlotte Gatherer, Leanne Walker, Frederick Russet, Veronique Humbertclaude, Gwen Dieleman, Katarina Dodig-Ćurković, Gaelle Hendrickx, Vlatka Kovač, Fiona McNicholas, Athanasios Maras, Santosh Paramala, Moli Paul, Ulrike M. E. Schulze, Giulia Signorini, Cathy Street, Priya Tah, Helena Tuomainen, Swaran P. Singh, Sabine Tremmery, Diane Purper-Ouakil

**Affiliations:** 1 CHU Montpellier-Saint Eloi, Médecine Psychologique de l’Enfant et de l’Adolescent, 80, Av Fliche, 34295 Montpellier Cedex 5, France; 2 Department of Child and Adolescent Psychiatry and Psychology, Erasmus University Medical Center, Rotterdam, Netherlands; 3 University of Osijek, Osijek, Croatia; 4 Department of Neurosciences, Child & Adolescent Psychiatry, University of Leuven, Leuven, Belgium; 5 Department of Child and Adolescent Psychiatry, School of Medicine and Medical Science and Geary Institute, University College Dublin, Dublin, Ireland; 6 Yulius Academy, Rotterdam, Netherlands; 7 Department of Child and Adolescent Psychiatry, Erasmus Medical Center-Sophia Children’s Hospital, Rotterdam, Netherlands; 8 Department of Child and Adolescent Psychiatry, Institute of Psychiatry, Psychology and Neuroscience, King’s College London, London, UK; 9 Centre for Interventional Paediatric Psychopharmacology and Rare Diseases (CIPPRD), National and Specialist Child and Adolescent Mental Health Services, Maudsley Hospital, London, UK; 10 HealthTracker Ltd, Gillingham, UK; 11 Mental Health and Wellbeing, Warwick Medical School, University of Warwick, Coventry, UK; 12 Stratford Child and Adolescent Health Service, Coventry and Warwickshire Partnership Trust, Stratford on Avon, UK; 13 Department of Child and Adolescent Psychiatry/Psychotherapy, University of Ulm, Ulm, Germany; 14 Psychiatric Epidemiology and Evaluation Unit, Saint John of God Clinical Research Centre, Brescia, Italy; 15 Department of Child & Adolescent Psychiatry, University Hospitals Leuven, Leuven, Belgium

## Abstract

**Background:**

Profound clinical, conceptual and ideological differences between child and adult mental health service models contribute to transition-related discontinuity of care. Many of these may be related to psychiatry training.

**Methods:**

A systematic review on General Adult Psychiatry (GAP) and Child and Adult Psychiatry (CAP) training in Europe, with a particular focus on transition as a theme in GAP and CAP training.

**Results:**

Thirty-four full-papers, six abstracts and seven additional full text documents were identified. Important variations between countries were found across several domains including assessment of trainees, clinical and educational supervision, psychotherapy training and continuing medical education. Three models of training were identified: i) a generalist common training programme; ii) totally separate training programmes; iii) mixed types. Only two national training programs (UK and Ireland) were identified to have addressed transition as a topic, both involving CAP exclusively.

**Conclusion:**

Three models of training in GAP and CAP across Europe are identified, suggesting that the harmonization is not yet realised and a possible barrier to improving transitional care. Training in transition has only recently been considered. It is timely, topical and important to develop evidence-based training approaches on transitional care across Europe into both CAP and GAP training.

**Electronic supplementary material:**

The online version of this article (10.1186/s12909-019-1576-0) contains supplementary material, which is available to authorized users.

## Background

Young people with psychological, emotional or behavioural problems who fall through the care gap when negotiating the transition boundary between child and adult mental health services are at risk of poorer mental health outcomes [1]. They may develop more serious mental disorders than those who experience a smooth and purposeful transition [1]. Referred to as “mental health service transition”, the move of young patients from child and adolescent mental health services (CAMHS) to adult mental health services (AMHS) is now understood to be more than an ‘event’ or a simple transfer [2]. It is a process, requiring therapeutic intent which prepares the adolescent for transition and includes a period of handover or joint care, transition-planning meetings and transfer of case notes or information summaries [3]. The importance of transitional care has been identified in young people with other chronic conditions such as congenital heart disease, juvenile arthritis, epilepsy and diabetes both at the individual and service provision levels [4–6].

Like the description of transition in physical medicine, transition in Mental Health Care (MHC) has multidimensional and multidisciplinary aspects [7–9]. The goal in transition is to maximize lifelong functioning and potential of young people through the provision of high-quality, developmentally appropriate health care that continues uninterrupted as the individual moves from adolescence to adulthood [8]. Appropriate transition is crucial for young service users and transition-related discontinuity of care is now considered as a major socioeconomic and societal challenge for the European Union (EU).

The EU-funded research program ‘MILESTONE project’ aims to improve transition of young patients from CAMHS to AMHS through a collaborative project involving eight different countries (www.milestone-transitionstudy.eu/) [10]. One of the MILESTONE work packages is specifically dedicated to training as a potential avenue for improvement. The rationale of this topic relies on previous findings having identified poor communication between CAHMS and AHMS, differences in care models and organization between CAHMS and AHMS, overload of services and absence of specific training as main obstacles to transition [11]. This has been confirmed by first findings from the MILESTONE consortium: Signorini and al. (2017) [12] identified lack of connection between CAMHS and AMHS, poor specific competencies and absence of systematic assessment and procedures for transitioning as targets for improvement. Implementation of specific evaluations and protocols will require specific training. Further training issues are the knowledge and skills required for planning transition, awareness of developmental needs, multidisciplinary collaboration and working with young people and families [1].

With the aim of developing and implementing training models and training procedures for clinicians across the EU, this work package is responsible for exploring how transition processes and outcomes might be related to the training of professionals working in mental health services in Europe. To the best of our knowledge, no study or review has addressed the training of professionals working in mental health services with a specific focus on transition between CAMHS and AMHS. As a prerequisite for further studies about relationships between transition and training, we conducted a systematic review of the structure and content of General and Adult Psychiatry (GAP) and Child and Adolescent Psychiatry (CAP) training in Europe. The aims were: 1/ to describe the organization and structure of current training across Europe in GAP and CAP, as defined by the European Union of Medical Specialists (UEMS) [13]; and 2/ to assess if and how transition is addressed in any of the European GAP and CAP training programs.

## Methods

The review was carried out following the standards of the Preferred Reporting Items for Systematic Reviews and Meta-Analyses (PRISMA) [14].

### Search strategy and data source

**Fig. 1:**
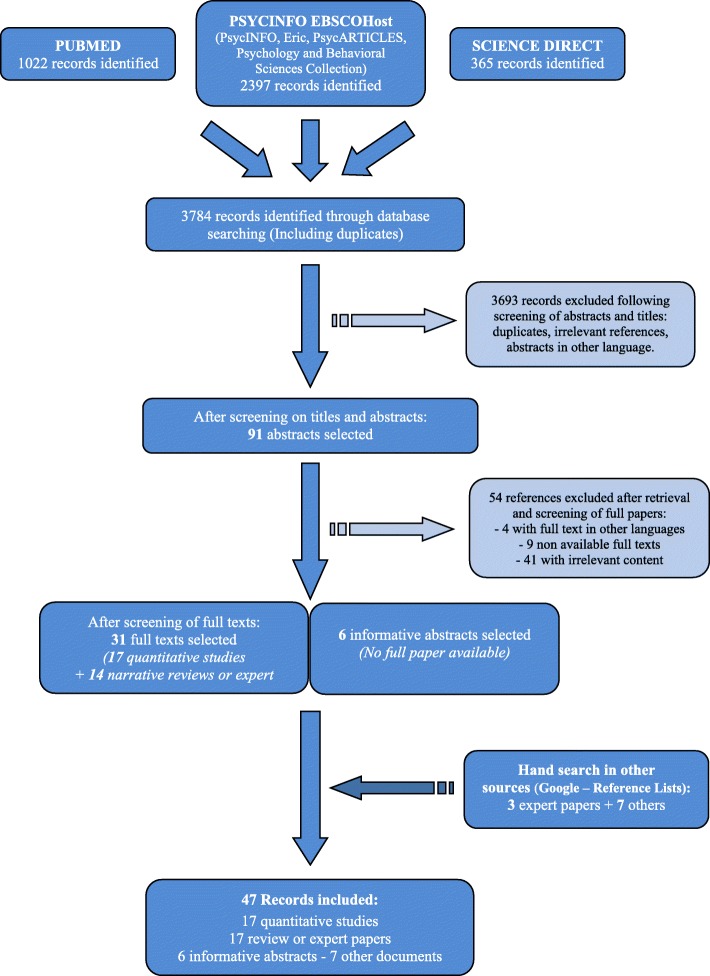
Flow chart: Systematic review on GAP and CAP Training in Europe

Two investigators (FR, VH) searched the literature through the following databases: PsycInfo-Esbco (Psycinfo, Eric, PsycARTICLES, Psychology and Behavioral Sciences Collection), PubMed and the revue collection Science Direct (Fig. 1). The review regarded organization and structure of training of CAP and GAP in Europe and was performed using subject headings with the following algorithm: (Psychiatr* AND Training AND Europ*); and (Psychiatr* AND Training AND [Name of 39 European countries]). These two algorithms were used in the following search sections: in “abstract” for PsycInfo-Esbco; in “title and abstract” for PubMed; in “Title, abstract and keywords” for Science Direct. Only references published after January 2000 were considered because there was no point in considering older articles due to reforms regularly implemented in psychiatry training. The search was conducted in March 2018.

The same investigators searched for the grey literature using various online sources for each country (e.g. medical associations, scientific societies, and universities), focusing on national psychiatry training programs and expert papers. Additional information was searched for by scanning the reference lists of identified papers. Authors were contacted if the full-text paper was not available.

### Data selection and quality assessment

The titles and abstracts of articles were screened for relevance by two reviewers (FR, AS). Potentially relevant articles were obtained in full and further screened to determine if they met the eligibility criteria detailed in Table 1. Authors were contacted when a full paper was not available. When a full paper was unavailable, the corresponding abstract was included if it provided relevant information about psychiatry training (Table 1). Expert papers, opinion papers and narrative reviews were included only if they met minimum standards according to JBI Critical Appraisal Checklist for Narrative, Expert Opinion and Text or JBI Critical Appraisal Checklist for Systematic Reviews and Research Syntheses [15] (Assessment Table available on request). For quantitative studies, the Hawker checklist [16] was used for critical appraisal. This checklist includes nine items. For every item, each study was given a quality score of 1 (very poor), 2 (poor), 3 (fair) or 4 (good), and finally granted a summed general score, with a maximum potential score of 36. Reviewers made their screening and assessments independently. In case of discrepancy in the screening, a consensus was searched. In case of discrepancy in the assessment, research papers were appraised jointly to reach an agreement.

**Table 1:** Eligibility criteria (Inclusion and exclusion criteria)

*Population*	Europe as a continent
	39 European countries *(Albania, Austria, Belgium, Bosnia, Bulgaria, Czech Republic, Cyprus, Croatia, Denmark, Estonia, Finland, France, Germany, Greece, Hungary, Ireland, Iceland, Italy, Latvia, Lithuania, Luxembourg, Malta, Moldavia, Montenegro, Netherlands, Norway, Poland, Portugal, Romania, Russia, Serbia, Slovakia, Slovenia, Spain, Sweden, Switzerland, Turkey, Ukraine, UK)*
*Interventions*	Psychiatry: General adult psychiatry, child and adolescent psychiatry, psychiatry in general or other psychiatry specialities if GAP or CAP were mentioned.
*Topic*	Training: all information related to specialist education after medical school (postgraduate training)
	Transition of patients from CAHMS to AHMS: identified as a process aiming to support young patients who move from CAHMS to AHMS - any kind of information concerning training to transition during specialist education.
*Study designs*	All types of studies: Reviews (systematic or narrative); Observational studies: surveys; Expert opinions; national programmes.
*Publications*	English, French or Spanish peer-reviewed journals
	Published from 01/01/2000

### Data extraction and analysis

Two data extraction forms (Additional file 1: Tables S1 & S2) were designed to collate information about GAP and CAP training for each European country according to the main aspects mentioned in the UEMS recommendations [13]: training program, structure and duration, quality control and assessment. Terms were defined according to the glossary established by UEMS [13] (Additional file 1: Appendix 1).

## Results

Forty-seven references were identified following the search regarding GAP and CAP training in Europe (Fig. 1). The quality scores of the 17 quantitative studies ranged from 17 to 30, out of a possible 36 (Table 2). The aims, methods and major aggregated data are described for full-text articles (Tables 3 and 4**)** and for abstracts (Table 5).

**Table 2:** Summary of quality score of the 17 quantitative studies

	Good (rated 4)	Fair (rated 3)	Poor (rated 2)	Very poor (rated 1)	Total rated
Abstract and title	9	4	0	4	17
Introduction and aims	3	11	3	0	17
Method and data	0	12	5	0	17
Sampling	2	6	9	0	17
Data analysis	3	2	1	11	17
Ethics and bias	2	4	6	5	17
Findings/results	12	6	0	0	17
Transferability/generalizability	0	12	5	0	17
Implications and usefulness	5	7	0	0	17
Distribution of Total score (max score = 36)	1 × 17 – 2 × 19 – 1 × 20 – 1 × 24 – 3 × 25 – 1 × 26 – 2 × 27 – 1 × 28 – 2 × 29 – 3 × 30

**Table 3:** Characteristics of selected full-papers: quantitative studies

Study	Aim	Methods	Results (aggregated data) and authors’ conclusion	Quality Score (/36)^a^
Margariti et al., 2002 [17]	To investigate the training in psychiatry provided in Greece in relation to the EBP recommendations	Quasi-experimental (quantitative) study: Structured questionnaire completed during an interview of the training directors of 14 institutions recognized bythe national authority as eligible to provide full-time training in psychiatry.	Response rate: 14/14 (100%) training directors.	30
			- Lack of a detailed national training plan	
			- The training provided shows great variability among institutions.	
			- Evaluation of the training programs not carried out by the national authority responsible for training centers (the Ministry of Health), leaving this task exclusively to the training centers themselves.	
Karabekiroglu et al., 2006 [18]	To provide a descriptive documentation on Child and Adolescent Psychiatry training in European countries	Quasi-experimental (quantitative) study: Survey. 10 questions sent by email to UEMS-CAP and EFPT representatives of 34 member countries of WHO -European region	Response rate: unknown/34 countries	28
			In 2006, European countries still have significant diversities in the structure of CAP training. There is still a long way to go for full harmonization across Europe.	
			- CAP is a known specialty in 23 countries and a subspecialty in 8 countries, but 5 countries do not have any structured CAP training. In 32.4% of the countries, CAP is not a specialty in its own right but is mostly linked to general psychiatry.	
			- After medical school, minimum training duration to become a CAP specialist: between 12 and 96 (mean: 59.71 ± 17.1) months.	
			- Only half of the countries have integrated a structured psychotherapy training in the programme	
			- More than two-thirds of the countries have started using logbooks to structure the curriculum.	
			- Around one-third of the countries have integrated structured research training into the CAP training programme.	
			- 37.9% of the countries: examination to begin CAP training. In 64.7%: examination to graduate. In 29.7% countries: both cases are reported.	
Lotz-Rambaldi et al., 2008 [19]	To evaluate the state of training in psychiatry in each member countries of UEMS and the current state of implementation of the UEMS recommended training requirements	Quasi-experimental (quantitative) study: Survey. Questionnaire: Part One to be completed by the national representative of each country in the EBP; Part Two to be completed by the chiefs of training and the representatives of trainees in training centres of the member states.	Response rate: Part One = 22/31 (71%) national representatives; Part Two = 409/923 (44%) questionnaires. Conclusion: The training requirements formulated by the EBP have been partly introduced in Europe (e.g. integration of psychotherapy) but the training in Europe is still very heterogeneous. - System of rotation not mandatory in most countries. - Areas of theoretical training (e.g. learning difficulties and mental handicaps) often not included in the compulsory common trunk of national training schemes. - No agreement within the EBP on the criteria for the definition of a sub-specialty.	28
Julyan, 2009 [20]	To make a point on educational supervision (ES) as an essential component of basic specialist training in psychiatry in the UK, with a focus on workplace-based Assessments (WPBA) as a new tool	Quasi-experimental (quantitative) study: Survey.	Response rate: Data 1 = 11 trainees and 11 supervisors (73%);	30
			Data 2 = 10 trainees and 10 supervisors (67%).	
			Conclusion: general agreement between trainees and supervisors, but some significant discrepancies.	
		All junior doctors and their educational supervisors in one UK psychiatry training scheme were surveyed both before (Data 1) and after (Data 2) the introduction of WPBAs		
			- Around 60% reported 1 h of ES per week or 3 times per month.	
			- ES was largely seen as useful.	
			- Around 50% of trainees and supervisors used 25–50% of ES time for WPBAs, with no impact on the usefulness of ES or the range of issues covered.	
			The impact of reduced training time, WPBAs and uncertainties over ES structure and content should be monitored to ensure that its benefits are maximized by remaining tailored to individual trainees’ needs.	
Kuzman et al. 2009 [21]	To evaluate the quality of the current residency training in psychiatry in Croatia using the subjective evaluations of the residency training that is being offered	Quasi-experimental (quantitative) study: Survey Questionnaire to residents from 15 Croatian psychiatric hospitals, clinics and wards in general hospitals	Response rate: 66/74 (89%) of all residents in September and October 2006 in Croatia. About a third of participants are only partially satisfied with the residency training that is being offered and its application in practice.	29
			They feel that most problems lie on the lack of practical psychotherapy, the inefficiency of the mentorship system and the lack of funding resources.	
Nawka et al. 2010 [22]	To present a trainee perspective on the major challenges in psychiatric training in Europe	Quantitative: Survey Survey of the 31 member countries of EFPT (trainees) about the 3 most important issues facing postgraduate training	Response rate: 28 /31 (90%) countries.	27
			Implementation of new postgraduate curricula in a number of countries (for example, the UK, Ireland, and the Netherlands)	
			- Insufficient training opportunities.	
			- Inadequate psychotherapy training.	
			Substantial differences in quality of training exist across Europe. Educational systems in some European countries have undergone major reforms.	
			Major concern reported by trainees: on the implementation of these new programs rather than to the structure or content of the curricula themselves.	
Oakley and Malik, 2010 [23]	To establish the variations in the pre-defined aspects of postgraduate psychiatric training within the member countries of the EFPT	Quantitative: Survey Structured questionnaire to delegates (trainees) at the EFPT 2008 forum	Response rate: 22/22 (100%) countries.	27
			Conclusion: The challenge of harmonizing training across Europe remains very real. - Wide variations in the length, content and structure of postgraduate psychiatric training across Europe.	
			- Some countries have no examinations or formal assessments, others have no compulsory placements.	
			- Five of the surveyed countries do not even have nationally standardized training schemes.	
			- Psychotherapy training is only compulsory in half the countries surveyed.	
Fiorillo et al., 2011 [24]	To explore training and practice of psychotherapy in ECPC members (countries of Northern, Southern and Western Europe)	Quasi-experimental (quantitative) study: Survey (Letter to editor) Online 16-item questionnaire on: quality of psychotherapy training, organizational aspects of psychotherapy training, satisfaction with training in psychotherapy, self-confidence in the use of psychotherapy	Response rate: 12/13 (92%) ECPC members.	30
			- Training in psychotherapy is mandatory in all of the 12 respondent countries, except Belgium and France.	
			- Training in psychodynamic and cognitive-behavioral therapies is available in almost all countries.	
			- Training in other therapies (systemic, interpersonal, supportive and psychoeducational, dialectical-behavioral) only in a few countries.	
			- Dedicated supervisor for training in psychotherapy not available in 5 countries out of 12.	
			- Psychotherapy competencies are evaluated differently, with no clear guidance regarding trainees’ evaluation in 15 countries.	
			Main barriers in accessing training in psychotherapy: difficulties to get time away from other duties, lack of supervisors, and lack of funding.	
Gómez-Beneyto et al., 2011 [25]	To know the psychiatry resident’s opinion and level of satisfaction on provided training	Quasi-experimental (quantitative) study: Survey Questionnaire to 363 trainees in 3rd and 4th year	Response rate: 216 (60%) residents.	24
			- The majority of residents had complied with the National Program for Psychiatric Training requirements.	
			- Level of satisfaction is fair.	
			- A small but substantial percentage did not comply adequately with the program, as regards: training in psychotherapy, research methodology, old age psychiatry, neurology and general medicine.	
Van Effenterre, 2011 [26]	To get an overview of trainees’ wishes as regards research training	Quasi-experimental (quantitative) study: Survey Questionnaire to members of the French association of trainees in psychiatry	Response rate: 45% trainees.	21
			- 25% of trainees achieved a research Master	
			- Lack of information on available possibilities in research during residency. Only 12% of residents think they were well informed. Tutorship would be a solution.	
Kuzman et al., 2012a [27]	To assess the problems in the implementation of psychiatric training curricula and the quality control mechanisms available in European countries	Quasi-experimental (quantitative) study: Survey (letter to editor)	Response rate: 29/ unknown total of countries	29
			- In 13 countries (45%), trainee representatives reported some differences between the psychiatric curriculum on paper and curriculum in practice	
		Representatives from EFPT member countries filled in a country report survey form. They were asked to rate the differences between the psychiatric curriculum on paper and the curriculum in practice in their countries as significant, existing to some extent or not existing. They were also asked to explain their understanding of such discrepancies in open ended questions		
			- In 9 countries (31%) significant differences were reported.	
			- In only 7 (24%) countries the curriculum was in line with training in practice.	
			- Placements considered as most problematic: psychotherapy (*n* = 13), research (*n* = 12) and addictions (*n* = 5).	
			- Most commonly reported reasons for discrepancies: lack of time for teaching activities (*n* = 11), lack of appropriate rewards for trainers (*n* = 9), lack of quality control measures (n = 9), and general shortage of supervisors (*n* = 7).	
			- In the countries with quality control (22/29), main mechanisms are: commissioned questionnaire reviews of placements, trainers/ supervisors and working conditions. Conclusion: several problems still influence the correct implementation of training curricula in practice. Establishing adequate quality control mechanisms for all national training programs is identified as one of the crucial steps in the improvement and harmonization of psychiatric training in Europe.	
Kuzman et al., 2012b [28]	To describe the structure and quality assurance mechanisms of post-graduate psychiatric training in Europe	Quasi-experimental (quantitative) study: Survey. Self-reported questionnaire completed by members of EFPT. The questionnaire consists of 20 questions: 10 on the structure of training program and the methods of assessment of trainees; 10 on the methods of quality assurance of the training programs. In order to ensure the reliability of the data, the respondents were asked to provide an official reference source) to be contacted in case of ambiguous responses.	Response rate: 29/ unknown total of countries	21
			Psychiatric training programmes and assessment methods are overall compatible in Europe but quality assurance mechanisms vary significantly.	
			- In 19/29 countries, the duration of the training programme is 5 years or more. - 26/29 countries have adapted a basic training programme that includes the ‘common trunk’ (according to UEMS definition) or a modified version of it.	
			- In 25/29 countries, trainees are evaluated several times during their training, with a final exam at the end.	
			- In 25/29 countries, official quality assurance mechanisms exist. However, results demonstrate great variations in their implementation.	
Simmons et al.,2012 [29]	To investigate trainee experiences of CAP training across Europe in 2010–2011 in three domains: structure and organization of training; training quality and content; and working conditions and recruitment	Quasi-experimental (quantitative) study: Survey Questions collated into a survey and addressed via email to CAP trainee representatives in 34 countries in Europe, using the EFPT email list	Response rate: 28/34 (82%) countries.	30
			Training experiences in CAP varies widely across Europe	
			- 7/28 countries (25%) have a core common trunk in general psychiatry before specialization in CAP.	
			- No official CAP training programme in 6/28 countries. Training standards are implemented in practice to a variable extent.	
			- In 19/28 countries (68%), supervision occurs at least weekly.	
			- Educational supervision is available in 13/28 countries (46%).	
			- Psychotherapy training is mandatory in 19/28 countries (68%).	
			- Research training is obligatory in 8/28 countries (29%).	
			- Subspecialty experience is extremely variable.	
Pinto Da Costa et al., 2013 [30]	To describe Portuguese psychiatry trainee’s opinion about their training and the modifications they would want to witness in the near future	Quasi-experimental (quantitative) study: Survey	Response rate: 80/193 (41.5%) psychiatry trainees.	29
			Changes claimed for: length and type of obligatory and optional placements, psychotherapy (who is obligatory in their training), easier access to research and clinical training opportunities abroad.	
		Structured questionnaire of 26 questions sent by email to Portuguese trainees		
Van Effenterre et al., 2013 [31]	To study the current situation of the academic training of French psychiatry trainees in psychotherapy during their residency	Quasi-experimental (quantitative) study: Survey Anonymous questionnaire sent to all French psychiatrist trainees through their local trainee association	Response rate: 869/1334 (65%) residents.	26
			- Training is insufficient for 75% trainees (much higher than in other countries).	
			- Different satisfaction rates across universities.	
			- Only 51% trainees have supervision, with large disparities between regions. All major therapies are represented.	
Van Effenterre et al., 2014 [32]	To study the teachers’ point of view on psychiatric training in France (weaknesses and strengths of the training, recent improvements and problems) and to compare with residents’ opinion	Quasi-experimental (quantitative) study: Survey	Response rate = 79/125 (63%) teachers.	29
		Emailed questionnaires sent in April 2012 to 125 academic professors and hospital practitioners (PU-PH)	- A majority of PU-PH (78%) willing to maintain a single training pathway including AP and CAP within a single diploma.	
			- Almost all suggested the implementation of an assessment of teaching and a formal mentorship program.	
			- Length of the training is a more controversial aspect.	
			- Suggested areas of improvement: training in psychotherapy and research, access to supervision. Crucial need to implement an efficient supervision during residency.	
Fàbrega Ribera & Ilzarbe, 2017 [33]	To evaluate the current situation experience of trainees interested in CAP involved in general psychiatry training.	Quasi-experimental (quantitative) study: Online survey	Response rate: 55/94 (59%) trainees	25
			- 4-month mandatory training in CAP included in the GAP programme	
		94 trainees identified as interested in working in CAP		
			- mandatory CAP placement	
			- CAP can also be a clinical elective rotation	
			- Time spent in CAP (mandatory placement + elective rotation): 3–20 months, median = 8 months	
			- Wide variability, from trainees being in CAP placements for 3 months to others being there for almost 2 years	

(^a^) See Table 2 for details

*Abbreviations*: *UEMS CAP* Union Européenne des Médecins Spécialistes section of Child and Adolescents Psychiatry, *EFPT* European Federation of Psychiatry Trainees, *WHO* World Health Organization, *WPA* World Psychiatric Association, *EBP* European Board of Psychiatry, *ECPC* Early Career Psychiatrists Committee, *CAP* Child and Adolescent Psychiatry, *AP* Adult Psychiatry, *ESCAP* European Society of Child and Adolescents Psychiatry, *GAP* General and Adult Psychiatry

**Table 4:** Characteristics of selected full-papers: expert opinion and narrative reviews

Study	Aim	Methods	Results (aggregated data) and authors’ conclusion	Quality Score (/36)
Hansen & Thomsen, 2000 [34]	To describe the structures of the Denmark and UK psychiatry training	Expert opinion	The UK postgraduate system puts greater emphasis on structuring the academic and clinical aspects of training.	NA
			The Danish system leaves the trainee in a more individualistic position.	
			Formalized training and supervision are sparse in Denmark compared with the UK. Some steps taken to harmonize the postgraduate psychiatric training of doctors in Europe. Still a very long way to go before trainees can move freely between EU countries with full recognition of their training.	
Furedi et al., 2006 [35]	To review the current status of psychiatry in selected countries of Central and Eastern Europe: Bulgaria, Croatia, Czech Republic, Hungary, Poland, Romania, Russia, Slovakia and Slovenia.	Narrative review. A group of psychiatrists from the region evaluated the status of psychiatry at the end of 2004 based on data from their countries and information available on WHO homepages	The systems of psychiatric training vary across the region but there is an effort to standardize national systems according to the WPA and UEMS requirements. Psychiatric training, pre-, postgraduate and continuous medical education are gradually being transformed.	NA
Zisook et al., 2007 [36]	To compare and contrast psychiatry residency training in the USA, in Canada and selected countries in South America (Chile, Brazil), Europe (UK, Sweden, Czech Republic), and Asia (India, Korea and China).	Expert opinion 9 individuals intimately familiar with psychiatry residency training in the USA, with prominent positions, and trained in other countries, describe their past training programs and make a comparison with USA training	Worldwide, psychiatry training varies considerably in different regions in terms of the duration of training, structure of clinical experiences, autonomy of trainee, didactic structure, level of supervision and rigor of evaluation. In some countries, training is much less structured than in the USA (e.g. Sweden). In others, it is somewhat more structured (e.g., Korea). Differences appear to be lessening.	NA
Naber & Hohangen, 2008 [37]	To describe training in psychiatry and psychotherapy in Germany	Expert opinion: Editorial	Since 1992, specialization in Germany is no longer in ‘psychiatry’ but in ‘psychiatry and psychotherapy’.	NA
			- Principal aim of training in Germany: to achieve a multidimensional approach to the diagnosis and treatment of psychiatric disorders.	
			- Special challenge: to offer psychotherapy training and to introduce psychotherapy into the classical spectrum of pharmaco and sociotherapeutic tools.	
			- Existing solution to face scarce funding for psychotherapy training: several hospitals providing a joint training programme for several psychiatry departments.	
Garret-Cloanec, 2010 [38]	Point of view about the current modifications of the Continuing Medical Education (CME) in France	Expert opinion: Editorial	The new system of CME in France is established by the law, based on the analysis of professional practice and the acquisition of knowledge or skills. Each professional must achieve his/her annual obligation by participating in one collective program. The organization is very complex, with the implications of many official organisms with various objectives. The lack of funding resources, with the possible intervention of pharmaceutical industries is also a problem.	NA
Javed et al., 2010 [39]	To describe the training and examination requirements of the new system in place in 2007 in the Psychiatric training in UK	Expert opinion	- The establishment of Postgraduate Medical Education and Training Board, Modernizing Medical Careers, new recruitment processes and changes in the curriculum and examination structure are all having a major impact on the future training and teaching programs in psychiatry in the UK. - Entry into psychiatry is becoming increasingly competitive and progression in career is now competency based in addition to the examination requirements subject to an annual review and regular appraisal. - A structured portfolio is also vital in order to present evidence of competencies and ensure smooth progression through the training grades.	NA
Bobes et al., 2012 [40]	To describe the current state of the Mental HealthCare Services in Spain	Narrative review A literature search performed using MEDLINE, Spanish journals, reference lists, national databases, and European and Spanish official documents	Specialist training programme in psychiatry was updated in 2008.	NA
			The new programme in psychiatry lasts four years.	
			Child and adolescent psychiatry is not recognized as a speciality.	
			Heterogeneous training of the specialists in charge of child and adolescent units is emphasized.	
Palha & Marques-Teixeira, 2012 [41]	To describe the panorama of psychiatry in Portugal, including training of professionals	Expert opinion	The rationale of the training is focused on the specificity of psychiatry on mental pathology, in the consequences of medical and chirurgical pathologies on the psychic system and in the always-considerable importance of the psychic system on the processes of human illness.	NA
			CAP is organized as an autonomous speciality (since 1959) with its specific training programme, rules and guidelines, as well as practice domain.	
Van Schijndel et al., 2012 [42]	To describe the state of psychiatry in the Netherlands	Expert opinion	Current programme developed and disseminated as from 1 January 2011.	NA
			- Backbone of the system: only one specialty with in-practice emphasis in three domains: CAP, AP or old age psychiatry, after a common trunk of general psychiatry.	
			- All the knowledge and the skills that should be achieved are described as competencies that are comprehensively assessed. - Trainees have an increased liberty to fill in their own preferences and tailor a training scheme based on their personal interests.	
Crommen, 2013 [43]	To present CAP in Belgium and the Flemish association for CAP	Expert opinion	The CAP training program consists of integrated theoretical, clinical and research components.	NA
			- Residents must complete at least 1 year of training in AP and at least 3 years of training in CAP during the 5-year program.	
			- Residents can also complete 1 year of pediatrics or neurology.	
			- Both the biological and the psychodynamic aspects of CAP are covered in the curriculum, and basic psychotherapy courses are provided.	
			Training program currently being revised for standardization with the UEMS.	
			No recognition of “child and adolescent psychiatry” as a medical specialty by the government in Belgium.	
Skokauskas, 2013 [44]	To review the current system of post-graduate training in psychiatry in Ireland	Comment on the National programme provided by the College of Psychiatry of Ireland	The current post-graduate training system aims to be in line with best European and International standards. - Length of post-graduate training: at least 7 years - Curriculum in two phases: Basic and Higher Specialist Training.	NA
			- Programme content and structure well defined.	
			- College of Psychiatry of Ireland: responsible for the training of specialists in psychiatry.	
Van Effenterre, 2013b [45]	To describe CAP training in France	Expert opinion	- One and only pathway for CAP and AP, leading to a generalist title of psychiatrist.	NA
			- 2 mandatory semesters in CAP for all trainees.	
			- Training program is not national but depending on universities and regions.	
Fegert et al., 2014 [46]	To describe CAP in Germany	Expert opinion: ESCAP Communication	In Germany, CAP first became an independent medical specialty in 1969.	NA
			The requirements for specialist training are currently under review by the authorities. Continuity of training is provided for and controlled by the “Continuous Medical Education System” (CME), according to which all child and adolescent psychiatrists must fulfill defined criteria for continuous field-related training within a 5-year period.	
Mayer et al., 2014 [47]	To compare the different curricula of post-graduate training in psychiatry in Europe	Narrative review of available publications on post-graduate training in psychiatry in Europe (Medline) + systematic overview for published postgraduate training curricula in Spanish, French, English and German (Goggle search) + e-mails sent to representatives of different professional medical societies	Medline search: 6 papers.	NA
			Google and personal contacts to representatives of professional medical societies: access and translation of original post-graduate curricula. Substantial differences between post-graduate training in the 6 European countries described (Germany, the Netherlands, Sweden, Belgium, France and UK): e.g. varying length, compulsory subjects, exam during training or final exam.	
Christodolou & Kasiakogia, 2015 [48]	To inform Greek psychiatrists and psychiatric trainees aspiring to emigrate in the UK. To describe the structure of the UK psychiatric training system and to compare it with the equivalent system in Greece	Expert opinion	Psychiatric training in the UK differs substantially to Greece in both structure and process:	NA
			- Pure psychiatric training in the UK Versus neurological and medical modules in Greece.	
			- In-training exams in the UK Versus only an exit exam in Greece - 3-year higher training in UK.	
Karwautz et al., 2015 [49]	To describe CAP in Austria	Expert opinion: ESCAP Communication	CAP specialty was established in 2007. From 2015, the training requirements are changing by law for all specialty fields.	NA
			In next curriculum: a 4-year phase of basic CAP training followed by three six-month modules focusing on specific topics like adolescent psychiatry, developmental psychiatry, addiction treatment or pediatric/psychosomatic medicine.	
Drobnic, 2016 [50]	A brief report about the state of CAP in Slovenia	Expert opinion: ESCAP Communication	In 2002, Slovenia started the first formal training in CAP. The training lasts 5 years, including 3 years of AP, 1.5 years of CAP, and 6 months of paediatrics and developmental neurology.	NA

*NA* non available, *UEMS CAP* Union Européenne des Médecins Spécialistes section of Child and Adolescents Psychiatry, *EFPT* European Federation of Psychiatry Trainees, *WHO* World Health Organization, *WPA* World Psychiatric Association, *EBP* European Board of Psychiatry, *ECPC* Early Career Psychiatrists Committee, *CAP* Child and Adolescent Psychiatry, *AP* Adult Psychiatry, *ESCAP* European Society of Child and Adolescents Psychiatry, *GAP* General and Adult Psychiatry

**Table 5:** Characteristics of selected abstracts

Study	Aim	Methods	Results (aggregated data) and authors’ conclusion	Quality Score (/36)
Buftea et al., 2010 [51]	To analyze the availability of types of psychotherapy and the commitment of psychiatry resident to psychotherapy training. Comparison with data from 1988	Quasi-experimental (quantitative) study: Survey	Response rate: unknown respondents / 728 (81.8% psychiatry residents). - Only 30.13% are involved in specific psychotherapy training, comparing with 48.5% in 1998.	NA
			- Available types of psychotherapy: CBT, positive psychotherapy, transactional analysis, psychoanalysis, psychodrama, hypnosis, existential psychotherapy.	
			- Even though training in psychotherapy has been a compulsory topic in curricula since 2007, its availability is still restricted, due to high costs, the need to self-finance the training, organizational difficulties and low number of training centers and trainers.	
Barrett et al., 2011 [52]	To gain insights regarding current CAP training within the member countries of the EFPT	Quasi-experimental (quantitative) study: Survey 10-item questionnaire to trainee representatives from 32 countries.	Response rate: 27 /32 (84.4%) respondent countries.	NA
			- In many countries, CAP and GAP training were not separate.	
			- In 35% of countries, CAP training was entirely separate from start of training.	
			- In 40%, entry to CAP training occurred after training in GAP.	
			- Variable availability of training posts.	
			- Varying duration of training: 3 years (19.2%), 4 years (23.1%), 5 years (26.9%).	
			Significant differences in CAP training experiences across 27 respondent countries.	
Giacco et al., 2011 [53]	To assess Early Career Psychiatrists’ (ECPs) satisfaction with training and self-confidence in different psychiatric domains; availability of clinical supervision and educational opportunities during training	Quasi-experimental (quantitative) study: Survey	Response rate: 194/ Unknown total respondents from 34 European countries	NA
		Online survey among European ECPs. self-reported questionnaires with multiple choice answers	- Most respondents (73%) were completely or partially satisfied with provided training.	
			- Most problematic areas: forensic psychiatry (68%), psychotherapy (63%) and CAP (57%).	
			- 30% of ECPs were not assigned to a tutor for clinical activities. - 67% did not receive any psychotherapeutic supervision.	
Kokras et al., 2011 [54]	To investigate, from a trainee’s point of view, the degree of compliance of Greek training centres to EBP recommendations	Quasi-experimental (quantitative) study: Survey Training centers in psychiatry were identified and trainees were invited by e-mail to complete an on-line survey in autumn 2010	Preliminary results from the first quarter of the sample.	NA
			- Vast majority of Greek psychiatric trainees do not have individualized training programs (88%) and logbooks (99%).	
			- No auditing experience (90%) and no exposure to internal (90%) or external (93%) evaluation.	
			- Structured theoretical training available to the majority of trainees (94%). - Only 25% are offered psychotherapeutic supervision.	
			Still inadequate compliance to some of the recommendations developed by the	
			EBP.	
Atti et al., 2012 [55]	To describe the opinion of Italian ECPs about provided training	Quasi-experimental (quantitative) study: Survey	Response rate: 244 respondents (216 last-year trainees and 8 recently qualified psychiatrists).	NA
		30-item questionnaire administered to all the participants during 3 years in a yearly training event for ECPs		
			- ECP felt the most uncomfortable in Forensic Psychiatry (62.5%), CAP (37.2%), and Dual Diagnosis/Substance-Abuse Related Disorders (33.9%).	
			- 45% of ECP complained that Psychotherapy is a critical issue.	
			- Though 46.4% of participants had supervision within the training program (less than two hours per week), the 87.4% sought help from external psychotherapeutic training programs.	
Lee & Noonan, 2012 [56]	To ascertain if trainees had fulfilled the Royal College of Psychiatrists’ psychotherapy training requirements, models of psychotherapy available and the availability of psychotherapy qualifications among consultants and senior registrars	Quasi-experimental (quantitative) study: Survey	Response rate: Unknown respondents / 62 (79%) registered college tutors.	NA
		A questionnaire was posted to all registered tutors in the Republic of Ireland	- No psychotherapy training was available according to 16.3% of tutors.	
			- Only 22.5% of tutors were aware of trainees who had met college training requirements in the previous two years.	
			- 79.8% of tutors reported that there were consultants and senior registrars with qualifications in psychotherapy.	
			Conclusions: Current training requirements are not being fulfilled. There are inadequate resources and time to formalise training. It is unlikely that the implementation of training requirements by the new college will be realisable without a review of training delivery.	

*UEMS CAP* Union Européenne des Médecins Spécialistes section of Child and Adolescents Psychiatry, *EFPT* European Federation of Psychiatry Trainees, *WHO* World Health Organization, *WPA* World Psychiatric Association, *EBP* European Board of Psychiatry, *ECPC* Early Career Psychiatrists Committee, *CAP* Child and Adolescent Psychiatry, *AP* Adult Psychiatry, *ESCAP* European Society of Child and Adolescents Psychiatry, *GAP* General and Adult Psychiatry

### General and adult psychiatry (GAP)

Available data regarding GAP training in Europe (absolute values and percentages) are summarized in Table 6 (detailed for each country in Additional file 2: Table S3 and Additional file 3:Table S4). Mentioned percentages were calculated on the total number of countries for which data were available. An established national standardized program with a mean duration of 4 to 6 years was evident in 76% of 34 European countries (data missing for Cyprus, Iceland, Luxembourg, Moldavia, Montenegro, and Ukraine). The compulsory and common set of fundamental knowledge for GAP, as defined by the UEMS [13] was largely followed by all countries, especially in relation to addictions (97% of countries), CAP (96%) and forensic psychiatry (88%), but less so in relation to old age psychiatry (69%) and psychotherapy (66%). In terms of common set of skills, placements in in-patient, outpatient and emergency psychiatry were compulsory in all countries, while training in liaison and consultation psychiatry was required in only 55% of countries. The evaluation of trainees varied considerably between countries, from a continuous examination during training by supervisor and workplace based-assessment (WPBA) to only a single final examination set by an examination board.

**Table 6:** General Adult Psychiatry training in Europe – synthesis of collected data

General Adult Psychiatry training	Number / number of known data (%)
National standardized training program	
Y	19 / 24 (79%)
Quality control of the training program	
Presence	
Y	20 / 23 (87%)
Realized by	
ministry of health or national board	16 / 23 (70%)
regional or university	3 / 23 (13%)
both	1 / 23 (4%)
Program length (years)	
< 4	3 / 34 (9%)
4 ≤ ≤ 6	26 / 34 (76%)
> 6	0 / 34
Contradictory	5 / 34 (15%)
Assessment	
Presence	
Y	25 / 32 (78%)
*during training*	*3 / 32 (9.5%)*
*during training and final board exam*	*19 / 32 (59%)*
*final board exam*	*3 / 32 (9.5%)*
N	1 / 32 (3%)
Contradictory	6 / 32 (19%)
Realized by	
supervisor only	10 / 28 (36%)
board commission	2 / 28 (7%)
WBA	4 / 28 (14%)
supervisor and board commission	6 / 28 (21%)
supervisor and WBA	5 / 28 (18%)
supervisor, board commission and WBA	1 / 28 (4%)
Logbook	
Y	23 / 28 (82%)
Consequences	
Y	18 / 25 (72%)
Compulsory common trunk of fundamental knowledge (UEMS 2003)	
General adult psychiatry	
Y	28 / 28 (100%)
CAP, learning difficulties and mental handicap	
Y	27 / 28 (96%)
Old age psychiatry	
Y	18 / 26 (69%)
Addictions	
Y	28 / 29 (97%)
Forensic psychiatry	
Y	23 / 26 (88%)
Psychotherapy	
Y	19 / 29 (66%)
N	7 / 29 (24%)
Contradictory	3 / 29 (10%)
Compulsory common trunk of skills (UEMS 2003)	
In-patient psychiatry	
Y	27 / 27 (100%)
Out-patient psychiatry	
Y	28 / 28 (100%)
Liaison and consultation psychiatry	
Y	15 / 27 (55%)
N	11 / 27 (41%)
Contradictory	1 / 27 (4%)
Emergency psychiatry	
Y	27 / 27 (100%)
Compulsory training	
Neurology	
Y	13 / 14 (93%)
Internal medicine	
Y	10 / 12 (83%)

*Abbreviations*: *Y* yes, *N* no, *WBA* workplace based assessment, *UEMS* Union Européenne des Médecins Spécialistes

The following items address relevant issues regarding training, but information was available only for some countries or was aggregated in publications:

* Structured theoretical training was provided in 18/22 (82%) countries [19], including Belgium, France, Germany, the Netherlands and Sweden [47], sometimes with variations within countries regarding implementation and content, for instance in France [57] or in Spain [25].
* Regarding Adult Psychiatry as a topic in the training program, the duration varied according to the training model used, with longer training in countries with separate specialities. Oakley & Malik [23] reported a mean training time of 23 months in 5 countries with a common training path (e.g. Bosnia, Croatia, Czech Republic, Estonia and France) compared to a mean training time of 39 months in 16 countries with separate training for adult psychiatry (e.g. Germany, Italy, UK).
* Training in psychodynamic psychotherapy, Cognitive Behavioural Therapy (CBT), family therapy and systemic therapy were the most widely available therapy courses [23, 24]. The implementation of training in psychotherapy has rarely been investigated. Lee and Noonan [56] conducted a survey among 62 registered college tutors in Ireland just prior to the College of Psychiatry of Ireland setting out psychotherapy training requirements: 16% of tutors reported that no psychotherapy training was available for the trainees, and only 22.5% of tutors were aware of trainees who had met college training requirements in the previous two years.
* There were major variations in supervision during speciality training, even within countries. Supervision was compulsory in Estonia [23], Germany [23, 47], Sweden [36, 47] and the UK [20]. It was optional in France [31] and Greece [54]. Data were contradictory for Italy: according to Oakley [23] it was compulsory for all, but Atti [55] indicated this to be the case for only 46% of trainees. Two types of supervision were commonly reported: clinical supervision in Estonia [23], Germany [23, 47], Sweden [36, 47], Greece [54], Italy [23], UK [58]; and educational supervision in Sweden [36, 47], UK [20] and Ireland [59]. Whatever the type of supervision, access was not always guaranteed for the trainees, evident in Italy [55] and Greece [54].
* Data about research training were scarce. Trainees in Belgium and the Netherlands were required to have at least one publication in a peer-reviewed journal [47]. Research training was optional in both France [26] and Portugal [30]. No scheduled time for research was available in Denmark [34].
* Continuing Medical Education (CME) was reported as mandatory in Germany [47], Hungary, Russia and Slovakia [35], France [36], with regular compulsory re-certification in Croatia [35], the Netherlands [47] and Poland [35]. This was also the case in Czech Republic but without specified sanctions in case of non-compliance [35]. CME was reported as optional in Bulgaria [35]; No data regarding CME was found for the other countries.

### Child and adolescent psychiatry (CAP)

Available data for CAP in each European country are summarized in Table 7 (detailed in Additional file 4: Table S5 and Additional file 5: Table S6*).* When aggregated data (not specifically detailed for each country) are reported in the text here below, the reference of the corresponding paper is provided.

**Table 7:** Child and Adolescent psychiatry training in Europe – synthesis of collected data

Child and Adolescent Psychiatry training	Number / number of known data (%)
Separate training of CAP and GAP	
Y	21 / 33 (64%)
*from start of training*	*18 / 33 (55%)*
*after common trunk with GAP*	*3 / 33 (9%)*
N	5 / 33 (15%)
Contradictory or not specified	7 / 33 (21%)
Is CAP a monospecialty / separate specialty?	
Y	24 / 34 (71%)
National training standards	
Y	26 / 29 (90%)
*fully implemented*	*10 / 29 (34%)*
*implemented in part*	*15 / 29 (52%)*
*not implemented*	*1 / 29 (4%)*
Is there a CAP theoretical program?	
Y	23 / 27 (85%)
*with standardized content*	*10 / 27 (37%)*
*without standardized content*	*13 / 27 (48%)*
N	3 / 27 (11%)
Contradictory	1 / 27 (4%)
Program length (total minimum after medical school to be a CAP specialist, in years)	
< 4	3 / 34 (9%)
4 ≤ ≤ 6	25 / 34 (73%)
> 6	4 / 34 (12%)
Contradictory	2 / 34 (6%)
Minimum duration specifically dedicated to CAP during this program (years)	
< 2	2 / 33 (6%)
2 ≤ < 3	10 / 33 (30%)
3 ≤ < 4	12 / 33 (36%)
≥ 4	4 / 33 (12%)
Contradictory	5 / 33 (15%)
Supervision	
Access to formal supervision	25 / 27 (93%)
Independent educational supervision	12 / 27 (44%)
Assessment	
Logbook	22 / 31 (71%)
Examination to be a trainee in CAP	12 / 30 (40%)
Examination to finish the training in CAP	22 / 34 (65%)
Duration of inpatient experience (months)	
< 12	5 / 27 (18%)
12 ≤ ≤ 24	11 / 27 (41%)
> 24	10 / 27 (37%)
Contradictory	1 / 27 (4%)
Duration of outpatient experience (months)	
< 12	8 / 27 (30%)
12 ≤ ≤ 24	11 / 27 (41%)
> 24	7 / 27 (26%)
Contradictory	1 / 27 (4%)
General adult psychiatry training	
Y	28 / 31 (91%)
*mandatory*	*26 / 31 (83%)*
*optional*	*2 / 31 (6%)*
N	1 / 31 (3%)
Contradictory	2 / 31 (6%)
Child neurology training	
Y	21 / 31 (68)
*mandatory*	*13 / 31 (42%)*
*optional*	*8 / 31 (26%)*
Not needed^a^	10 / 31 (32%)
Paediatric experience	
Y	21 / 33 (64%)
*mandatory*	*14 / 33 (43%)*
*optional*	*3 / 33 (9%)*
*not specified*	*4 / 33 (12%)*
Not needed^a^	7 / 33 (21%)
Contradictory	5 / 33 (15%)
Neurology experience	
Y	16 / 27 (59%)
*mandatory*	*4 / 16 (25%)*
*optional*	*2 / 16 (12.5%)*
*not specified*	*10 / 16 (62.5%)*
N	10 / 27 (37%)
Contradictory	1 / 27 (4%)
Psychotherapy training	
Presence	
Y	20 / 34 (59%)
*mandatory*	*14 / 34 (41%)*
*optional*	*6 / 34 (18%)*
Not needed^a^	7 / 34 (20.5%)
Contradictory	7 / 34 (20.5%)
Program structure	
Theoretical & practical	24 / 26 (92%)
Theoretical only	4 / 26 (8%)
Type	
CBT	21 / 21 (100%)
systemic	17 / 21 (81%)
psychodynamic	18 / 21 (86%)
other	5 / 21 (24%)
Is research experience compulsory?	
Y	6 / 33 (18%)
N	19 / 33 (58%)
Contradictory	8 / 33 (24%)

*Abbreviations*: *Y* yes, *N* no, *CAP* child and adolescent psychiatry, *GAP* general adult psychiatry, *CBT* cognitive behavioural therapy

^a^: may correspond to ‘no’ or ‘yes optional’

CAP and GAP training were delivered separately at the start of training in more than half of the European countries; yet for a further 21% of countries, this relationship could not be precisely determined due to contradictory data available (Czech Republic, Estonia, Latvia and UK) **(**Additional file 4: Table S5) or because the term “separate” is not defined in the available literature. The case of Spain is particular: CAP and GAP are now separated according to the Law but this separation has not been implemented yet, leading to still one common training [33].

Out of the 27 countries for which data was provided, 85 % of European countries had a standardized CAP theoretical program. However, fully implemented national training standards were identified in only 34% of countries. The total length of training required for becoming a CAP specialist ranged from 4 to 6 years for 73% of countries, including a period between 2 and 4 years dedicated specifically to CAP for 66% of these countries.

In CAP training, the mean duration of compulsory time spent in CAP and Adult Psychiatry (AP) placements was 38 months and 13 months, respectively [23]. Training and experience in child neurology, paediatrics and neurology were required in 42, 64 and 59% of countries, respectively, but content and duration were rarely specified for these topics. More details were available concerning psychotherapy. Training in psychotherapy involved theory and practice in 92% of countries. It included CBT in 100% of countries and systemic and psychodynamic therapies in respectively 81 and 86%. It was mandatory in 41% of countries.

Supervision was available in 93% of countries. As regards the types of supervision, trainees had access to independent educational supervision in only 44% of countries. No data were found for other types (e.g. clinical supervision). Frequency of supervision varied between countries: it occurred weekly in 19/28 (68%) countries, or alternatively daily or every few months [29]. Research experience was not compulsory in 58% of the countries.

Finally, assessment methods varied widely, with oral exams in 19/28 (68%), workplace assessments in 16/28 (57%) and written exams in 12/28 (43%) countries [29]. Compared with GAP, no data about CME was available, except for Germany [46].

### Transition in GAP and CAP programmes

Only two countries mentioned transition in their programmes: UK and Ireland. However, no specific mention was found in the GAP section: either for Ireland, or for UK in the Curriculum for Specialist Training in General Psychiatry [58] or in the Curriculum for the Core Training [60]. Transition was only addressed in CAP programmes. In the UK, it was mentioned in the Curriculum for Specialist Training in CAP (ST4–6/Higher training) [61]. In the mandatory part of the training, it was briefly referred to in connection with assessing and managing main clinical diagnoses in adolescence and future outcomes, and working in collaboration with children/young people and families and appropriate teams. The skills section was more specific and outlined a practical step to be taken when facilitating transitioning care from CAMHS, i.e. preparing transition plans taking account of local protocols. An optional learning objective linked with transition was also available, providing more details regarding various aspects of transition. In Ireland, transition was mentioned briefly in the CAP section of the Curriculum for basic and higher specialist training in psychiatry [59], advising that Case Based Discussion should be applied when managing transition of an adolescent to an adult mental health service.

## Discussion

The aim of this review was to determine current training programmes in General Adult Psychiatry and Child and Adolescent Psychiatry across Europe and to assess if and how transition as a topic is incorporated in the training curricula of these disciplines. A systematic review was conducted and provided 45 documents.

### Current GAP and CAP training in Europe: the issue of harmonization

A key objective of the European Economic Community is to allow the free movement of professionals (Treaty of Rome, 1957). Hence, one of the major challenges concerning psychiatry in Europe has been the harmonization of training and certification requirements. Various professional organizations have been working for 20 years on recommendations to harmonize an optimal quality of national psychiatry training programs in Europe [62, 63].

According to data collected through our review, this harmonization has reached a significant level on several aspects of both GAP and CAP trainings: national programs, program length (in the average range of 4–6 years for about 3/4 of countries), mandatory GAP training in CAP and mandatory CAP training in GAP. For GAP training in particular, quality control of the training programs implementation is reported in most countries. For CAP, supervision in general is widely accessible in the vast majority of countries.

However, harmonization is still to be achieved on several other aspects. Most importantly, in both CAP and GAP trainings, differences between the stated national programmes and the lived experience of trainees are reported, suggesting substantial variations at a local level [20, 29, 63]. For both, there is still no final board exam in 1/3 of countries and no mandatory training in psychotherapy in 1/3 or even more for CAP. As regards data available for GAP more specifically, crucial aspects of the compulsory common trunk of knowledge and skills defined by UEMS [13] like old age psychiatry or liaison and consultation psychiatry are still not mandatory (respectively, in 31 and 41% of countries)^1^^,^^2^. In terms of examination and assessment methods, there is still no consequence in case of failed assessment in 28% of countries. Concerning assessment methods in particular, a most interesting evolution is taking place but is still restricted to a few countries like UK [64] or the Netherlands [42]: a shift from a system based only on participation of the trainee towards a “competence-based training” model where trainees are much more responsible and skills are central. Finally, very scarce data about CME show that few countries keep a register or set minimum standards [65] and that there are important variations in modalities [35, 36, 47]. For CAP, available data show harmonization is not yet realised regarding the access to an independent educational supervision.

**Fig. 2:**
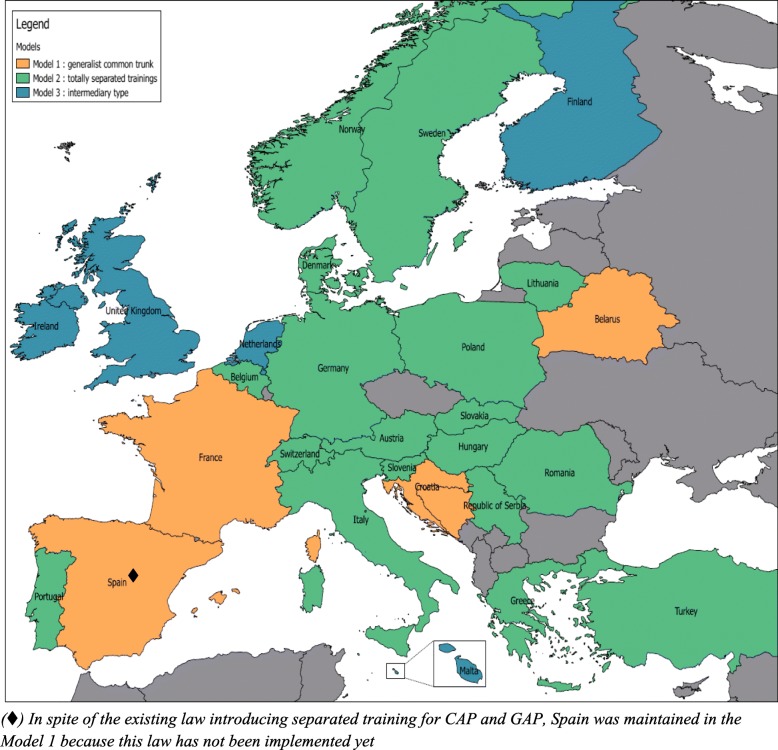
Mapping of 3 coexisting models of training and practice of GAP and CAP in Europe

As a major manifestation of this harmonization still to be achieved, we delineated 3 coexisting models of training and practice of GAP and CAP in Europe (Fig. 2). In the first model, psychiatry is a general speciality, with possible subspecialties that are not mandatory. Trainees are provided with a generalist education and receive a general diploma of “psychiatrists”. This model was identified only in 5/33 countries (15%)^3^, among which Spain was included since the separation of GAP and CAP has not been implemented in the training programmes yet [33]. In the second model, psychiatry is divided into totally independent specialties (e.g. CAP, AP, forensic, addictions, old age, etc). Trainees are provided with a separate specialized training from the start after medical studies, with completely different programs. This model is common and prevalent in 18/33 countries (55%)^4^. Finally, 5/33 countries (15%)^5^ countries used a third model, where trainees were provided with a common specialist psychiatry core program followed by further specialization – this often led to longer total training periods. Five remaining countries (15%)^6^ could not be classified, due to unclear or contradictory data.

### Transition as a topic in training of CAP and GAP in Europe

This review identified only two countries where this topic appeared in the curricula. In Ireland and the UK, transition has recently become a mandatory topic, but it is only covered briefly in the training documentation (in the UK, however, an elective course provides also more detailed training). Furthermore, transition is only addressed in CAP training, with no mention of it in GAP training. Likewise, training in transition has been newly identified by the UEMS as part of the goals that should be acquired by trainees, but this is limited to CAP in the interim [66, 67]. It is important to note that both Ireland and the UK fall into the third model, which involves long periods in training and possibly allows for varied topics to be covered, including transition as a topic.

Outside Europe, authors from Australia, Canada and the United States (US) have identified difficulties in access to care and coordinated services for youth with mental health conditions [68–71]. Training in transitional care has also been identified as a strategy to aid continuity of care and support for different domains of functioning in young people with mental health conditions. Cross-training about transition in mental health where adult and child case managers are trained together has been documented in 19 out of 50 states in the US [72] and a systems of care guidelines for transition-aged youth has been provided [73].

Within the MILESTONE-project training material about transition in mental health conditions will be delivered. These training modules are intended for health care professionals, stake-holders and for the general public and will be made available on the project website: www.milestone-transitionstudy.eu. In France, a specific training module has recently been added in the revised mandatory national curriculum of trainees in psychiatry https://sides.uness.fr/.

### Facilitators and/or barriers to transition in training - avenues

Relationships between GAP and CAP are a crucial issue in the transition process, both in terms of the experience that professionals have of the other discipline and in terms of common knowledge allowing a better dialogue and collaboration. Data collected in this review are reassuring from this point of view: 83% (26/31) of CAP training programs required a compulsory period of training in GAP and 96% (27/28) of GAP training programs required a compulsory period of training in CAP (Tables 6 and 7). However, the real length of exposure to the other discipline and content of training is variable and should be specifically explored in further studies.

Crucial structural differences in training models should be taken into account, as they probably have an impact on the relationships between both specialities. Thus, the monospeciality type of training (model 2) may significantly reduce the training in CAP for those who choose GAP specialty, and vice versa. Separate training pathways with no common basis, and often with no training provided in the other speciality (e.g. Germany), may contribute to a fragmented understanding of, and less experience in developmental psychopathology. While adolescence is a crucial period in the emergence of psychopathology and onset of disorders, trainees undergoing GAP training in this model may end up with a lack of knowledge and understanding of developmental psychopathology. The generalist type of training (model 1) and the common core program with further mandatory specialization (model 3) appear to better guarantee a more balanced experience in both specialities during the whole postgraduate training. These two models may also guarantee a better cooperation between child and adult psychiatrists when young service users face transition. The generalist training should, nevertheless, be long enough to allow a real core training in which CAP occupies a significant part of the curriculum. In its publications, the MILESTONE group have started examining the influence of the different training models on the transition outcome of young people in the MILESTONE study, combining European mapping data of child and adolescent mental health services with data on training models [74].

### How can training programs ensure improved quality of transition?

First, in order to improve the quality of care in transition, both CAP and AP training programs should definitely start including transition as a mandatory subject. This is the direction currently followed by professional organizations: the two corresponding sections of UEMS have recently been involved in discussions regarding transition and a joint working group has been set up to look at transition from child to adult services [66, 74]. Fegert et al. (2017) mention “transition psychiatry” as a topic to be established both in training and continuous medical education, to compensate for a missing expertise [67, 75].

Second, the content of training must be reviewed. A structured and evidence-based training to transition, related to skills, should be provided as a priority. The TRACK study [1] suggested four major criteria for an optimal transition in mental health care, which could act as a starting point for training in transition: 1) ‘Continuity of care’, 2) ‘Period of parallel care (relational continuity)’, i.e. a period of joint working where the service user is involved with both CAMHS and AMHS; 3) ‘Transition planning meetings’ (cross-boundary and team continuity)’, i.e. at least one meeting discussing the transition from CAMHS to AMHS, involving the service user and/or carer and key professionals, prior to the handover of care from CAMHS to AMHS; 4) ‘Optimal information transfer (information continuity)’, i.e. referral letter, summary of CAMHS contact, any or all CAMHS notes and a contemporary risk assessment.

Beyond the transition as a topic in itself, developing other specific related topics is also crucial: 1) promoting a life span concept of the patient, like in the USA where training about child and adult development is available during the core training [36]; 2) extending this developmental approach particularly in GAP training [8, 76, 77], giving trainees mandatory experience across ages [64]. This is particularly needed for neurodevelopmental disorders like Attention-Deficit Hyperactivity Disorder [78] or Autism Spectrum Disorder, which are now well known to go on far beyond childhood and adolescence. 3) The specific needs and issues of adolescents and young adults should also be emphasized, as has already been done for the elderly in many countries (without necessarily making it a specialty in itself). The care for young people should be more comprehensive, or far-reaching, and take into account potential school problems, autonomy, support and involvement of parents, professional involvement, all of which necessitate collaboration between professionals and developing partnerships. For many years now, somatic medicine has emphasised this necessary focus on adolescents and their specific needs [7, 9, 77, 79]. A position paper about transitional care in adolescents with chronic conditions published by the Society of Adolescent Medicine has identified environmental support, decision-making and consent, family support and professional sensitivity to psychosocial issues as key factors for a successful transition [80]. Therefore, training in transition care should not only be a symptoms-based approach but a comprehensive developmental approach. Health providers in both paediatric and adult settings should be trained in shared case management. Contents of training should include the development of decision-making skills in adolescents during the transition process as well as family support because some parents will need the help of health providers to adjust to the changing needs of their children. In a study about parent perspectives, family members of young people with mental health conditions requested service providers to consider them as resources and potential collaborators in supporting young people in transition to live successful lives in the community [81].

Third, what is the best timing and manner for delivering training on transition and other relevant topics? A minimum mandatory content regarding transition should be included in training (in theoretical courses or in case studies). CME could be another opportunity for training in transition, provided that relevant modules are available. Developing a CME training programme in transition is one of the objectives of the MILESTONE Project. Joint training events between CAMHS and AMHS professionals could also be an avenue, particularly because they have been shown to improve working relationships and create opportunities for collaborative work [82–84].

### Limitations

As regards the critical analysis of papers selected in our systematic review, several aspects should be taken into account when considering the results. Half of the references were expert papers, narrative reviews and other documents of the grey literature. Therefore, our review suffers from limitations usually related to the very nature of these documents (e.g. non-representative samples of studies, lack of quality appraisal, and multiple citation bias). The formal quality appraisal of the quantitative studies yielded low scores in sampling and data analysis as well as in ethics/bias assessment. Transferability/generalizability was questionable due to the type of data collection. Indeed, trainees were the only source of information in a majority of the studies (17/22, 77%). This participant selection may have impacted the quality of the reported information because trainees may not be aware of every aspects of the official curricula and may rely on limited experience within their own training centres. Our review also suffers from database and language biases because certain national journals may have been underrepresented in the databases we used and because of exclusion of a limited amount of studies in other languages that English or Spanish.

Some data were not available for all countries, making comparisons difficult when exploring the harmonization process. In addition, the number of references on CAP and GAP training varied widely between countries, from one to more than six references per discipline and country. Most countries (68% for CAP and 86% for GAP) had between two to five references per discipline (Additional file 2: Table S3, Additional file 3: Table S4, Additional file 4: Table S5 and Additional file 5: Table S6). More specifically, scarce (information obtained for < 5/19 items) or no data was available for 9/35^1^ countries regarding GAP, and for 6/35^2^ countries regarding CAP. Some essential issues were poorly covered: minimum length of training in psychotherapy, research, exact content of theoretical education, time dedicated to the different disciplines, continuing medical education.

Contradictory data were found in 5/19 items for GAP training and 10/19 items for CAP training (e.g. duration of GAP training in France was six years according to Kuzman [28] and four years according to Mayer [47]). This was particularly significant (with more than 10% of contradictory data) for program length (GAP and CAP training), assessment, and mandatory psychotherapy (GAP training), separate GAP and CAP training, paediatric experience, and psychotherapy training (CAP training) (Additional file 2: Table S3, Additional file 3: Table S4, Additional file 4: Table S5 and Additional file 5: Table S6). Lack of precise definitions of terms used in questionnaires may have contributed to this, leading to unclear or imprecise questions and/or answers (e.g. what is meant by the word “speciality”, or “separate training”). *Another limitation is that* the degree of implementation of national programs was not measurable, as this aspect were not systematically analysed at both national and local levels.

Finally, this review focusses on psychiatry but it must be kept in mind that other mental health disciplines are also involved in transition (i.e. psychologists, behavioural therapists and psychotherapists, psychiatric nurses, paediatricians). The training of professionals in these disciplines should be explored in relation with transition as well.

### Strengths

Our systematic review of literature aimed at minimizing bias and reducing subjectivity by usage of inclusion/exclusion criteria and of a formal quality appraisal. Interpretation bias was also limited by the different background of the researchers. Among the twenty-three questionnaire-based surveys (abstracts and full text altogether) seventeen reported response rates (Tables 3, 4 and 5), most of which can be considered good: between 60 and 80% in 6/16 (37%) surveys and equal or more than 80% in 7/16 (44%).

## Conclusion

To the best of our knowledge, this is the first systematic review on General Adult Psychiatry and Child and Adolescent Psychiatry training in Europe. It is also the first to focus on transition as a topic in training programs. Three coexisting models of training in GAP and CAP across Europe have been identified, indicative of a harmonization still in progress. As these three models do not allow the same level of collaboration between GAP and CAP professionals, it is a possible barrier to a good quality or smooth transition. Future studies on the content, quality and experience of training should focus on including all key stakeholders involved in psychiatry programs (i.e. not only trainees, but also early career psychiatrists, professors and representatives of national professional organisations). Although probably a crucial element for ensuring optimal transition of mental health service users, transition as a topic in training is only beginning to be considered. We have suggested several avenues to foster this trend. The increasing common reflection between both specialities and sub-specialities is a promising move towards further positive developments.

## Additional file

Additional file 1: **Appendix 1** Glossary, according to UEMS, 2003. **Table S1.** Data extraction form elaborated for adult psychiatry training. **Table S2.** Data extraction form elaborated for child and adolescent psychiatry training. (DOC 100 kb)

Additional file 2: **Table S3.** General and adult psychiatry training in Europe - Available data detailed for each country (1). (XLS 39 kb)

Additional file 3: **Table S4.** General and adult psychiatry training in Europe - Available data detailed for each country (2). (XLS 40 kb)

Additional file 4: **Table S5.** Child and adolescent psychiatry training in Europe - Available data detailed for each country (1). (XLS 41 kb)

Additional file 5: **Table S6.** Child and adolescent psychiatry training in Europe - Available data detailed for each country (2). (XLS 39 kb)

## Abbreviations

AMHS: Adult Mental Health Service
AP: Adult Psychiatry
CAMHS: Child and Adolescent Mental Health Service
CAP: Child and Adolescent Psychiatry
CBT: Cognitive Behavioural Therapy
CME: Continuing Medical Education
EU: European Union
GAP: General and Adult Psychiatry
MHC: Mental Health Care
PRISMA: Preferred Reporting Items for Systematic Reviews and Meta-Analyses
UEMS: European Union of Medical Specialists

## Footnotes

1 Albania, Moldavia, Luxembourg, Cyprus, Montenegro, Iceland, Bulgaria, Hungary, Norway.

2 Moldavia, Luxembourg, Cyprus, Montenegro, Ukraine, Malta.

3 Belarus, Bosnia, Croatia, France and Spain.

4 Austria, Belgium, Denmark, Germany, Greece, Hungary, Italy, Lithuania, Norway, Poland, Portugal, Romania, Serbia, Slovakia, Slovenia, Sweden, Switzerland, Turkey.

5 Finland, Ireland, Malta, the Netherlands, UK.

6 Bulgaria, Czech Republic, Estonia, Latvia, Russia.

## References

[1] Singh SP, Paul M, Ford T, Kramer T, Weaver T, McLaren S (2010). Process, outcome and experience of transition from child to adult mental health care: a multiperspective study. Br J Psychiatry.

[2] Brodie I, Goldman R, Clapton J (2011, Revised 2014). Mental health service transitions for young people. Research briefing 37. Social Care Institute for Excellence www.scie.org.uk/publications/briefings.

[3] Paul M, Street C, Wheeler N, Singh SP (2015). Transition to adult services for young people with mental health needs: a systematic review. Clin Child Psychol Psychiatry.

[4] Agarwal SD, Barnett ML, Souza J, Landon BE (2018). Adoption of Medicare’s transitional care management and chronic care management codes in primary care. JAMA.

[5] Burke L, Kirkham J, Arnott J, Gray V, Peak M, Beresford MW (2018). The transition of adolescents with juvenile idiopathic arthritis or epilepsy from paediatric health-care services to adult health-care services: a scoping review of the literature and a synthesis of the evidence. J Child Health Care.

[6] Wright C, Steinway C, Jan S (2018). The genesis of systems of care for transition to adulthood services: emerging models in primary and subspecialty care. Curr Opin Pediatr.

[7] Royal College of Paediatrics and Child Health (2003). Bridging the gaps: healthcare for adolescents.

[8] Health and Social Care Advisory Service (HASCAS) (2006). HASCAS tools for transition - CAMHS to adult transition. A literature review for informed practice.

[9] McDonagh JE, Viner RM (2006). Lost in transition? Between paediatric and adult services. BMJ.

[10] Singh SP, Tuomainen H, de Girolamo G, Maras A, Santosh P, et al. Protocol for a cohort study of adolescent mental health service users with a nested cluster-randomised controlled trial to assess the clinical and cost effectiveness of managed transition in improving transitions from child to adult mental health services (The MILESTONE study). BMJ Open. 2017. 10.1136/bmjopen-2017-016055. 10.1136/bmjopen-2017-016055 PMC5652531 29042376

[11] Singh SP (2009). Transition of care from child to adult mental health services: the great divide. Curr Opin Psychiatry.

[12] Signorini Giulia, Singh Swaran P., Marsanic Vlatka Boricevic, Dieleman Gwen, Dodig-Ćurković Katarina, Franic Tomislav, Gerritsen Suzanne E., Griffin James, Maras Athanasios, McNicholas Fiona, O’Hara Lesley, Purper-Ouakil Diane, Paul Moli, Russet Frederick, Santosh Paramala, Schulze Ulrike, Street Cathy, Tremmery Sabine, Tuomainen Helena, Verhulst Frank, Warwick Jane, de Girolamo Giovanni (2018). The interface between child/adolescent and adult mental health services: results from a European 28-country survey. European Child & Adolescent Psychiatry.

[13] Union Européenne des Médecins Spécialistes (UEMS), Section for Psychiatry - European Board of Psychiatry (2017). Charter on training of medical specialist in the EU - Requirements for the speciality of psychiatry.

[14] Liberati A, Altman DG, Tetzlaff J, Mulrow C, Gøtzsche PC, Ioannidis JPA (2009). The PRISMA statement for reporting systematic reviews and meta-analyses of studies that evaluate health care interventions: explanation and elaboration. Plos Med.

[15] Joanna Briggs Institute (2016). Critical appraisal tools.

[16] Hawker S, Payne S, Kerr C, Hardey M, Powell J (2002). Appraising the evidence: reviewing disparate data systematically. Qual Health Res.

[17] Margariti M, Kontaxakis VP, Madianos M, Feretopoulos G, Kollias K (2002). Psychiatric education: a survey of Greek trainee psychiatrists. Med Educ.

[18] Karabekiroglu K, Doğangün B, Hergüner S, von Salis T, Rothenberger A (2006). Child and adolescent psychiatry training in Europe: differences and challenges in harmonization. Eur Child Adolesc Psychiatry.

[19] Lotz-Rambaldi W, Schafer I, ten Doesschate R, Hohagen F (2008). Specialist training in psychiatry in Europe--results of the UEMS-survey. Eur Psychiatry.

[20] Julyan TE (2009). Educational supervision and the impact of workplace-based assessments: a survey of psychiatry trainees and their supervisors. BMC Med Educ.

[21] Kuzman MR, Jovanović N, Vidović D, Margetić BA, Mayer N, Plestina S (2009). Problems in the current psychiatry residency training program in Croatia: residents’ perspective. Coll Antropol.

[22] Nawka A, Kuzman MR, Giacco D, Malik A (2010). Mental health reforms in Europe: challenges of postgraduate psychiatric training in Europe: a trainee perspective. Psychiatr Serv.

[23] Oakley C, Malik A (2010). Psychiatric training in Europe. Psychiatrist.

[24] Fiorillo A, Luciano M, Giacco D, Del Vecchio V, Baldass N, Oakley C (2011). Training and practice of psychotherapy in Europe: results of a survey. World Psychiatry.

[25] Gomez-Beneyto M, Montilla-García JF, De Castro-Manglano P, Gay-Pamos E, González-Torres MA, Vallejo-Ruiloba J (2011). (2011) *La opinión de los residentes de psiquiatría sobre la formación que reciben.* (Spanish). The opinion of psychiatric residents on the training they receive. Actas Esp Psiquiatr.

[26] Van Effenterre A (2011). Formation et information des internes en psychiatrie: quelle place pour la recherche ? (French) education and training of young psychiatrists: is there time for research?. Encephale.

[27] Kuzman MR, Giacco D, Simmons M, Wuyts P, Bausch-Becker N, Favre G, Nawka A (2012). Are there differences between training curricula on paper and in practice? Views of European trainees. World Psychiatry.

[28] Kuzman MR, Giacco D, Simmons M, Wuyts P, Bausch-Becker N, Favre G, Nawka A (2012). Psychiatry training in Europe: views from the trenches. Med Teach.

[29] Simmons M, Barrett E, Wilkinson P, Pacherova L (2012). Trainee experiences of child and adolescent psychiatry (CAP) training in Europe: 2010-2011 survey of the European Federation of Psychiatric Trainees (EFPT) CAP working group. Eur Child Adolesc Psychiatry.

[30] Pinto da Costa M, Guerra C, Malta R, Moura M, Carvalho S, Mendonça D (2013). Psychiatry training towards a global future: trainees’ perspective in Portugal. Acta Med Port.

[31] Van Effenterre A, Azoulay M, Champion F, Briffault X (2013). Initial training in psychotherapy for psychiatrists in France: results of a national survey. Encephale.

[32] Van Effenterre A, Hanon C, Llorca PM (2014). (Enquête auprès des PU-PH Sur la formation en psychiatrie en France.) survey among academic teachers about psychiatric training in France. Encephale.

[33] Fabrega M, Ilzarbe D (2017). Becoming a child and adolescent psychiatrist in Spain: trainees’ perspectives. Eur Child Adolesc Psychiatry.

[34] Hansen LK, Thomsen AF (2000). Psychiatric training in two different EU countries: Denmark and the UK. Psychiatrist.

[35] Füredi J, Mohr P, Swingler D, Bitter I, Gheorghe MD, Sartorius N (2006). Psychiatry in selected countries of central and Eastern Europe: an overview of the current situation. Acta Psychiatr Scand.

[36] Zisook S, Balon R, Björkstén KS, Everall I, Dunn L, Yoo T (2007). Psychiatry residency training around the world. Acad Psychiatry.

[37] Naber D, Hohagen F (2008). Training in psychiatry and psychotherapy in Germany. Encephale.

[38] Garret-Gloanec, N. (2010). La FMC est-elle DPC au profit de l'EPP ? (French) L’Information Psychiatrique, 86(5), 379–383. doi: 10.1684/ipe.2010.0642.

[39] Javed MA, Ramji MA, Jackson R (2010). The changing face of psychiatry training in the UK. Indian J Psychiatry.

[40] Bobes J, Garcia-Portilla MP, Bobes-Bascaran MT, Parellada M, Bascaran MT, Arango C (2012). The state of psychiatry in Spain. Int Rev Psychiatry.

[41] Palha A, Marques-Teixeira J (2012). The emergence of psychiatry in Portugal: from its roots to now. Int Rev Psychiatry.

[42] Van Schijndel MA, Gerrits WL, Niesink P, van der Gaag RJ (2012). The state of psychiatry in the Netherlands: strength by quality, influence by capabilities. Int Rev Psychiatry.

[43] Crommen S (2013). Child and adolescent psychiatry in Belgium and the Flemish Association for Child and Adolescent Psychiatry. Eur Child Adolesc Psychiatry.

[44] Skokauskas N (2013). Postgraduate Training in Psychiatry in Ireland.

[45] Van Effenterre A (2013). Formation en pédopsychiatrie en France: historique, actualités et réflexions. Inf Psychiatr.

[46] Fegert J, Schepker R, Banaschewski T, Flechtner H (2014). Child and adolescent psychiatry in Germany. Eur Child Adolesc Psychiatry.

[47] Mayer S, van der Gaag RJ, Dom G, Wassermann D, Gaebel W, Falkai P, Schüle C, European Psychiatric Association (2014). European psychiatric association (EPA) guidance on post-graduate psychiatric training in Europe. Eur Psychiatry.

[48] Christodoulou N, Kasiakogia K (2015). Psychiatry training in the United Kingdom -part 2: the training process. Psykiatriki.

[49] Karwautz A, Purtscher-Penz AK, Hochgatterer P, Kienbacher C, Board of the Austrian Society of Child and Adolescent Psychiatry (2015). Child and adolescent psychiatry in Austria. Eur Child Adolesc Psychiatry.

[50] Drobnic M (2016). The state of child and adolescent psychiatry in Slovenia: a brief report. Eur Child Adoles Psychiatry.

[51] Buftea L, Cretu C, Mihai A (2010). 10 years re-evaluation of number of psychiatric residents involved in training in psychotherapy in Romania. Eur Psychiatry.

[52] Barrett EP, Nawka A, Malik A, Giacco D, Rojnic-Kuzman M, Simmons M, Favre G (2011). Child and adolescent psychiatry training in Europe: Views of trainee representatives for 2009–2010 to the European Federation of Psychiatric Trainees. Eur Psychiatry.

[53] Giacco D (2011). ECP09-05 - psychiatric training in europe: the opinions of early career psychiatrists. Eur Psychiatry.

[54] Kokras N., Samiotakis G., Gerasi E., Oikonomou D., Ntoumanis A., Psarras R. (2011). P03-560 - A survey on psychiatric training in greece from trainees’ perspective. European Psychiatry.

[55] Atti AR, Forlani M, Morri M, Fiorillo A, Volpe U, De Rosa C, Ferrari S (2012). Quality of training program for early-career psychiatrists in Italy: focus on forensic psychiatry and psychotherapy. Eur Psychiatry.

[56] Lee A, Noonan A (2012). Psychotherapy training in Ireland: a survey of college tutors. Ir J Psychol Med.

[57] Bulletin Officiel N°39 du 28 octobre 2004. From https://www.affep.fr/formation/formation.php.

[58] Royal College of Psychiatrists (2010, Revised 2016). A competency based curriculum for specialist training in psychiatry. Specialists in General Psychiatry. https://www.rcpsych.ac.uk/pdf/General_Psychiatry_Curriculum_August_2016.pdf.

[59] College of Psychiatry of Ireland (2016). Curriculum for basic and higher specialist training in psychiatry.

[60] Royal College of Psychiatrists (2013, updated 2016). A Competency Based Curriculum for Specialist Core Training in Psychiatry. Core training in Psychiatry CT1-CT3. https://www.rcpsych.ac.uk/pdf/Core_Psychiatry_Curriculum_August_2016.pdf.

[61] Royal College of Psychiatrists (2013). A competency based curriculum for specialist training in psychiatry. Specialists in Child and Adolescent Psychiatry. https://www.gmc-uk.org/-/media/documents/Amended_Child_and_Adolescent_Curriculum_January_2018.pdf_72924518.pdf.

[62] Hill P, Rothenberger A (2005). Can we - and should we have a europsychiatry for children and adolescents? The work of the UEMS section and board for child and adolescent psychiatry/psychotherapy. Eur Child Adolesc Psychiatry.

[63] Brittlebank A, Hermans M, Bhugra D, Pinto Da Costa M, Rojnic-Kuzman M, Van der Gaag RJ (2016). Training in psychiatry throughout Europe. Eur Arch Psychiatry Clin Neurosci.

[64] Weerasekera P, Feinstein RE (2013). The future of psychiatric education: an international perspective. Acad Psychiatry.

[65] Muijen M (2010). Training psychiatrists in Europe: fit for purpose? Commentary on... Psychiatric training in Europe. Psychiatric Bull.

[66] Union Européenne des Médecins Spécialistes, Section of Psychiatry (2014). Annual Report.

[67] Fegert et al. (2016) Transition from adolescence to adulthood: the challenges to establish “transition psychiatry”. https://www.escap.eu/care/transition-to-adulthood-and-effective-mental-health-care/transition-key-issues-paper.

[68] Carver J, Cappelli M, Davidson S, Caldwell W, Belair MA, Vloet M (2015). Taking the next step forward: building a responsive mental health and addictions system for emerging adults.

[69] McGorry P, Bates T, Birchwood M (2013). Designing youth mental health services for the 21st century: examples from Australia, Ireland and the UK. Br J Psychiatry.

[70] Pottick KJ (2008). US patterns of mental health service utilization for transition-age youth and young adults. J Behav Health Serv Res.

[71] Nguyen T, Embrett MG, Barr NG, Mulvale GM, Vania DK, Randall GE, DiRezze B (2017). Preventing youth from falling through the cracks between child/adolescent and adult mental health services: a systematic review of models of care. Community Ment Health J.

[72] Davis M, Sondheimer DL (2005). State child mental health efforts to support youth in transition to adulthood. J Behav Health Serv Res.

[73] Clark HB, Davis M (2000). Transition to adulthood: a resource for assisting young people with emotional or behavioral difficulties.

[74] Singh SP, Tuomainen H, de Girolamo G, et al. Protocol for a cohort study of adolescent mental health service users with a nested cluster-randomised controlled trial to assess the clinical and cost effectiveness of managed transition in improving transitions from child to adult mental health services (The MILESTONE study). BMJ Open. 2017. 10.1136/bmjopen-2017-016055. 10.1136/bmjopen-2017-016055 PMC5652531 29042376

[75] Union Européenne des Médecins Spécialistes, Section of Psychiatry (2016). Annual Report.

[76] Lamb C, Hall D, Kelvin R, Van Beinum M. Working at the CAMHS/adult Interface: good practice guidance for the provision of psychiatrists services to adolescents/young adults. London: Royal College of Psychiatrists; 2008.

[77] Society for Adolescent Medicine (2003). Transition to adult health care for adolescents and young adults with chronic conditions. Position paper for the Society of Adolescent Medicine. J Adolesc Health.

[78] Hall CL, Newell K, Taylor J, Sayal K, Swift KD, Hollis C (2013). ‘Mind the gap’ – mapping services for young people with ADHD transitioning from child to adult mental health services. BMC Psychiatry.

[79] Bartsocas C (2007). From adolescence to adulthood: the transition from child to adult care. Health Deliv.

[80] Blum RW, Garell D, Hodgman CH, Jorissen TW, Okinow NA, Orr DP, Slap GB (1993). Transition from child-centered to adult health-care systems for adolescents with chronic conditions: a position paper of the Society for Adolescent Medicine. J Adolesc Health.

[81] Jivanjee P, Kruzich JM, Gordon LJ (2009). The age of uncertainty: parent perspectives on the transitions of young people with mental health difficulties to adulthood. J Child Fam Stud.

[82] Lamb C, Murphy M (2013). The divide between child and adult mental health services: points for debate. BJP.

[83] Bruce H, Evans N (2008). The transition into adult care. Psychiatry.

[84] Moscoso A, Jovanovic N, Rojnic M (2015). Transition from adolescent to adult mental health services in Europe from the provider's perspective. Lancet Psychiatry.

