# Supplementary material for: Training of adult psychiatrists and child and adolescent psychiatrists in europe: a systematic review of training characteristics and transition from child/adolescent to adult mental health services
Source: BMC Med Educ. 2019 Jun 13;19:204. doi: 10.1186/s12909-019-1576-0 (PMC6567390; doi:10.1186/s12909-019-1576-0)
Supplement: Supplementary file 1 — Appendix 1 Glossary, according to UEMS, 2003. Table S1. Data extraction form elaborated for adult psychiatry training. Table S2. Data extraction form elaborated for child and adolescent psychiatry training. (DOC 100 kb) [file 12909_2019_1576_MOESM1_ESM.doc]

**Appendix 1** – **Glossary, according to UEMS, 2003 (Training of medical specialists on the EU – Requirements for the specialty of psychiatry – 11.10.2003)**

Common trunk: That compulsory part of the educational input that is fundamental and shared by all trainees.

Developmental psychiatry: That area of psychiatry concerned with the normal and abnormal development of the child. It involves assessing and studying the interplay of biological, family, social and other environmental factors. Developmental Psychiatry is normally considered to apply until early adulthood. It is not a defined clinical subspecialty but rather an area of research and theoretical understanding.

General adult psychiatry: Psychiatry concerned with mainstream adult psychiatric disorder and excluding child and adolescent psychiatry, old age psychiatry, mental handicap (learning disability) psychiatry, substance misuse psychiatry and forensic psychiatry.

Supervision (clinical): Advice on routine management issues with a focus on the care of individual patients, e.g. as part of a ward round, community team discussion or out-patient clinic discussion of cases.

Supervision (educational): Regular, weekly, individual or small group discussion of topics, relating to clinical work, professional development from a general prospective, interpersonal work-related issues, career advice, discussion of academic issues raised by routine clinical work and exam practice.

**Table S1** – Data extraction form elaborated for adult psychiatry training

| **General Adult Psychiatry training** | Mandatory training scheme *(generalist / monospecialties / common trunk + advanced specialties / None compulsory)* | | |
| --- | --- | --- | --- |
|  | National standardized program *(Yes / No)* | | |
|  | Quality control of the training program *(Yes, ministry of health or national board / Y, regional or university / No)* | | |
|  | Program structure *(theoretical only / practical only / theoretical and practical / If theoretical, is the content standardised?)* | | |
|  | Program length *(number of mandatory years)* | | |
|  | Compulsory common trunk of fundamental knowledge (according to UEMS 1997) | General adult psychiatry (*Yes / No)* | |
|  |  | Old age psychiatry *(Yes / No)* | |
|  |  | Addictions *(Yes / No)* | |
|  |  | CAP, learning difficulties and mental handicap *(Yes / No)* | |
|  |  | Forensic psychiatry *(Yes / No)* | |
|  |  | Psychotherapy *(Yes / No)* | |
|  | Compulsory training in neurology *(Yes / No)* | | |
|  | Compulsory training in internal medicine *(Yes / No)* | | |
|  | *Optional subjects /subspecialisations (adult psychiatry / CAP / old age psychiatry / addiction and substance misuse / forensic / emergency / liaison psychiatry / mental handicap and learning disabilities / neurology / paediatrics / none compulsory)* | | |
|  | Theoretical training: total number of hours | | |
|  | Placements / Practical training | Compulsory common trunk of skills (according to UEMS 1997) | In-patient psychiatry (short, medium and long stay)  *(Yes / No)* |
|  |  |  | Out-patient psychiatry (community psychiatry, day-hospital)  *(Yes / No)* |
|  |  |  | Liaison and consultation psychiatry  *(Yes / No)* |
|  |  |  | Emergency psychiatry  *(Yes / No)* |
|  |  | Total number of placements | |
|  |  | Duration *(number of hours)* | |
|  | Psychotherapy | Psychotherapy *(Yes mandatory / Yes optional / No)* | |
|  |  | Program structure *(theoretical only / practical only / theoretical and practical)* | |
|  |  | Type *(psychodynamics / CBT / systemic / psychoeducation / cognitive remediation)* | |
|  |  | Duration *(number of hours)* | |
|  | Research | Research (*Yes mandatory / Yes optional / No*) | |
|  |  | Type *(theoretical training only / practical experience only / theoretical and practical)* | |
|  |  | Duration *(number of hours)* | |
|  | Supervision | Supervision *(Yes mandatory / Yes optional / No)* | |
|  |  | Type *(educational / clinical / psychotherapy)* | |
|  |  | Duration *(number of hours)* | |
|  | Assessment | Assessment *(Yes / No)* | |
|  |  | Realised by *(supervisor / board commission / workplace-based assessments)* | |
|  |  | Logbook *(Yes / No)* | |
|  |  | Consequences *(Yes / No)* | |
|  | Final/graduate national board examination *(Yes / No)* | | |
|  | Continuous education | Continuous education *(Yes mandatory / Yes optional / No)* | |
|  |  | Regular recertification required by the law *(Yes / No)* | |
|  | Transition | *Yes mentioned / No mention* | |

**Table S2** – Data extraction form elaborated for child and adolescent psychiatry training

| **Child and Adolescent Psychiatry Training** | Separate training of CAP and AP/GAP *(Yes / No)* | |
| --- | --- | --- |
|  | Is CAP a monospecialty / separate specialty? (*Yes / No)* | |
|  | National standardized program *(Y, fully implemented / Y, implemented in part /Y, not implemented / N)* | |
|  | Quality control of the training program *(Yes, ministry of health or national board / Y, regional or university / No)* | |
|  | Program structure *(theoretical only / practical only / theoretical and practical / If theoretical, is the content standardised?)* | |
|  | Program length: *minimum in CAP / total minimum after medical school to be a CAP specialist (months)* | |
|  | Theoretical training: total number of hours | |
|  | Placements / Practical training | Mandatory placements *(psychiatric university hospital / psychiatric non-university hospital / general hospital / private practice / community health centre / in-patient long stay / day hospital / None compulsory)* |
|  |  | Total number of placements |
|  |  | Duration *(number of hours)* |
|  |  | Duration of inpatient / outpatient experience *(months)* |
|  | Child neurology training *(Yes mandatory / Yes optional / No)* | |
|  | Paediatric experience *(Yes mandatory / Yes optional / No)* | |
|  | Neurology experience *(Yes mandatory / Yes optional / No)* | |
|  | General / adult psychiatry training *(Yes mandatory / Yes optional / No)* | |
|  | Psychotherapy | Psychotherapy (*Yes mandatory / Yes optional / No)* |
|  |  | Program structure *(theoretical only / practical only / theoretical and practical)* |
|  |  | Type *(psychodynamics / CBT / systemic / psychoeducation / cognitive remediation)* |
|  |  | Duration *(number of hours)* |
|  | Research | Research *(Yes mandatory / Yes optional / No)* |
|  |  | Type *(theoretical training only /practical experience only / theoretical and practical)* |
|  |  | Duration *(number of hours)* |
|  | Supervision | Supervision: *(Yes mandatory / Yes optional / No)* |
|  |  | Type *(educational / clinical / psychotherapy*) |
|  |  | Duration *(number of hours)* |
|  | Assessment | Assessment *(Yes / No)* |
|  |  | Realised by *(supervisor / board commission / workplace-based assessments)* |
|  |  | Logbook *(Yes / No)* |
|  |  | Consequences *(Yes / No)* |
|  | Is there any examination to be a trainee in CAP? *(Yes / No)* | |
|  | Final/graduate national board examination *(Yes / No)* | |
|  | Continuous education | Continuous education *(Yes mandatory / Yes optional / No)* |
|  |  | Regular recertification required by the law *(Yes / No)* |
|  | Transition | *Yes mentioned / No mention* |
